# Lessons Learned From A Geriatric Referral Model in People With HIV

**DOI:** 10.1093/ofid/ofaf661

**Published:** 2025-10-23

**Authors:** Sarah R Gorvetzian, Kristine M Erlandson, Evelyn Iriarte, Skotti Church, Jacob Walker

**Affiliations:** Department of Medicine, University of Colorado, Aurora, Colorado, USA; Department of Medicine, University of Colorado, Aurora, Colorado, USA; College of Nursing, University of Colorado, Aurora, Colorado, USA; Department of Medicine, University of Colorado, Aurora, Colorado, USA; Department of Medicine, University of Colorado, Aurora, Colorado, USA

**Keywords:** care model, comprehensive geriatric assessment, geriatrician, HIV provider

## Abstract

People with HIV experience geriatric syndromes commonly found in much older populations and may benefit from geriatrician consultation. Multiple clinical models have emerged to address this need. We describe a model at the University of Colorado and the barriers to its robust implementation. The UCHealth Infectious Disease clinic implemented a referral-based model to connect high-risk people with HIV ≥50 years old with the UCHealth Seniors clinic. During geriatric consultation, patients were screened for functional status, preventative care, socioenvironmental factors, and cognitive/mental health concerns. Geriatricians communicated recommendations to the primary HIV provider. HIV providers completed a survey about the program. From January 2018 to July 2019, 11 patients underwent geriatric consultation. The average age was 69 years old (SD = 7.4; range 59–80). Geriatricians made recommendations regarding referral to other health or community-based services (*n* = 9), medication changes (*n* = 8), osteoporosis screening (*n* = 6), and management of memory problems (*n* = 5). Advanced directives were discussed in all visits. Almost all primary HIV providers said they would refer future patients to the program. The geriatric consultation program improved access to geriatric care but with limited uptake. We posit that lack of provider and patient consensus on the utility of geriatric care, the lack of a co-located clinical model, challenges in addressing polypharmacy, geriatrician shortages, and financial constraints all act as barriers to implementation of models of HIV geriatric care.

## Teaser

We describe a geriatric consultation model for older adults with HIV and its limited uptake in a US clinic. Despite several positive outcomes, implementation barriers—including structural, financial, and workforce challenges—highlight the need for integrated, scalable models of HIV-geriatrics care.

Over half of the overall population of people with HIV in the US and Europe is now aged 50 years or older. As a result of multiple factors, including HIV-associated inflammation, long-term side effects of antiretroviral therapy, and socioeconomic factors, many people with HIV are experiencing syndromes commonly found in much older patients, including falls, incontinence, dementia, frailty, cardiovascular disease, and impairment in activities of daily living [1, 2]. Studies show that people with HIV experience both a higher prevalence of geriatric syndromes (ie, “accentuated aging”) as well as earlier onset of these syndromes (ie, “accelerated aging”) when compared with people without HIV [3–5]. As people with HIV become older, many may benefit from a consultation with a geriatrician. Geriatricians, physicians with additional training and expertise in the care of older adults, can offer advice on the management of geriatric syndromes, understanding goals of care, and minimizing medical interventions and treatments that may not prolong the health span [6]. Optimal strategies for implementing geriatric care in this population are not yet clear.

Multiple institutions across the US and the UK have developed clinic models to address the unique needs of the aging population of people with HIV [7]. Examples of existing models involve a consulting geriatrician via referral from an HIV provider, a joint clinic model with both HIV and geriatric providers, or a provider dually-trained in geriatrics and HIV medicine [8]. Some clinic models do not involve a geriatrician at all, and instead employ nurse-lead teams or a nurse specialist trained in geriatrics [8]. In 2018, the UCHealth Infectious Disease Clinic at the University of Colorado, Aurora, implemented an outpatient referral program called the Positive Aging Consultation program to connect high-risk older people with HIV with specialty geriatric care. Despite a relatively large pool of eligible patients, there was limited utilization of the geriatric consultation model. The purpose of this article is to describe the structure of this clinical model and discuss potential barriers that may have prevented higher participation in the program. Exploration of these barriers can provide key insights into how to improve access to geriatric care for this population.

## MATERIALS & METHODS

### Setting and Population

The UCHealth Infectious Disease Clinic is an outpatient infectious disease practice located within the University of Colorado, a tertiary care center located in Aurora, CO, USA. The clinic serves as a medical home for more than 2000 people with HIV, is supported by funding from the Ryan White HIV/AIDS Program and is staffed by University of Colorado School of Medicine faculty. Within the UCHealth Infectious Disease Clinic, people with HIV who are ≥50 years old were considered for referral to the Seniors Clinic, an outpatient geriatric primary care practice located on a different floor of the same building as the Infectious Disease clinic. Typically, patients must be ≥75 years of age to be seen in the Seniors Clinic, but an exception was made for the patients participating in the program [9]. During the trial period, the Infectious Disease Clinic had 903 people with HIV ≥50 years old who were eligible to access the Positive Aging Consultation program. There were no additional exclusion criteria.

### Procedures

#### Positive Aging Consultation Program

HIV providers were encouraged to refer patients who were considered high-risk, including those with geriatric syndromes (eg, vision loss, hearing loss, urinary incontinence, mobility issues, falls history, inability to perform activities of daily living [ADLs], and inability to perform instrumental activities of daily living [IADLs]), hyper-polypharmacy (defined as taking ≥10 medications, inclusive of antiretroviral therapy) [10], multimorbidity (defined as ≥2 long-term health conditions in one individual [11]), or in need of assistance with advance care planning. Recruitment of patients occurred via emails to HIV providers, posters in the UCHealth Infectious Disease Clinic, and discussion during clinic staff meetings. Ultimately, referral to the Positive Aging Consultation program was up to the HIV provider's clinical discretion. Referrals were sent via email or through an order in the electronic health record. Additionally, pharmacy residents screened all 903 eligible patients and identified patients taking ≥10 medications and contacted them by phone or via the electronic patient portal to offer Positive Aging Consultation program visits.

During all visits to the Positive Aging Consultation program, patients were screened for four major categories including functional status, preventative care, socioenvironmental factors, and cognitive concerns/mental health using a standardized provider note template created by the consulting geriatricians with input from an HIV provider. While the provider template included prompts for the formal screening tools described in Table 1, utilization of these tools was not required for completion of a comprehensive geriatric assessment and was ultimately left to the geriatric provider's discretion.

**Table 1.** Four Major Categories of Assessment During Geriatric Consultation Visit

	Functional Status	Preventative Care Screenings	Socioenvironmental Factors	Cognition
**Assessment tools**	Patient reports of: • Vision loss • Hearing loss • Urinary incontinence • Mobility issues • Use of mobility aids • Falls in preceding year • Ability to perform standardized list of activities of daily living (ADL's) and instrumental activities of daily living (IADL's)[12] Timed get-up-and-go test[13] Grip strength testing[14]	Chart review for completion of: • Routine preventative care screenings, (colon, breast, and cervical cancer) • Osteoporosis screening • Nutrition screening/weight changes • Routine vaccinations	Patient reports of: • Living situation • Food access • Transportation • Social supports • Social isolation • Elder abuse • End of life planning	Cognition screening tools: • Montreal Cognitive Assessment (MoCA)[15] • Mini-Cog[16] Mood disorder screening tools: • Depression screening—Patient Health Questionnaire (PHQ-2)[17] • Anxiety screening—General Anxiety Disorder-7 (GAD-7)[18]

The consulting geriatrician made clinical recommendations, including referrals, imaging studies, or alterations to medications, either via communication with the primary provider or by directly placing orders. The means by which recommendations were made was decided at the discretion of the geriatric provider depending on the nature of the recommendation and clinical context. HIV medications were not altered by the geriatric provider. Patients returned to the care of their primary HIV provider at the end of consultative services. Initial geriatrics consult visits were 60 minutes in length; 40-minute follow-up visits were scheduled at the recommendation of the geriatrician.

Payment for geriatric consultation was done through patient's insurance and was billed as a new patient consult visit (Current Procedural Technology [CPT®] codes 99204, 99205) [19].

#### Clinical Pharmacy Consultation

Patients taking ≥10 medications were referred for clinical pharmacy consultation, to be completed prior to the Positive Aging Consultation appointment. Patients referred for pharmacy consultation were instructed to bring all medications, both prescribed and over the counter, to their appointment with an infectious disease pharmacist. Visits were conducted within the HIV clinic. Pharmacists performed a detailed assessment of the patient's current medications, with special attention to drug-drug interactions, medications that could potentially be discontinued, or potentially inappropriate medications as identified by Beers Criteria (an explicit list from the American Geriatric Society of potentially inappropriate medications that should typically be avoided in older individuals) [20]. These recommendations were available to the geriatrician to incorporate into the consult visit. All patients who underwent clinical pharmacy consultation also underwent geriatrician consultation.

### Data Collection

Written consent was obtained from patients and providers to participate in data collection and follow-up surveys. Data was collected via chart review of all geriatric and pharmacy notes, referrals placed, and follow-up notes with the primary HIV provider or other members of the interdisciplinary team. In addition to functional status, preventative care, socioenvironmental factors, and cognitive/mental health concerns described above, data collection included demographic data (gender, race, ethnicity, and membership in a vulnerable group defined as prisoner, cognitively impaired, other), general medical data (vital signs, number of hospitalizations, number of HIV visits in preceding year, substance use, comorbidities), and HIV status (year of diagnosis, most recent CD4 count and viral load, FIB-4 score, Veterans Aging Cohort Study [VACS] index score, and current HIV medications). A 5-month chart review was performed to document primary provider visits since geriatric consultation and implementation of geriatrician recommendations. Data was recorded in a secure REDCap database. Primary HIV providers were asked to perform a follow-up survey of the Senior Clinic visit, which included overall satisfaction with the geriatric and pharmacy consultation, likelihood of implementing consultant recommendations, and likelihood of referring patients to the program in the future. Analysis of the data was primarily descriptive. Means and standard deviation were used for the description of numerical data elements. Colorado Multiple Institutional Review Board approval was obtained for this study under protocol number 17–2160.

## RESULTS

### Participants

Between January 2018 to July 2019, of the 903 patients eligible for the referral program, 11 patients were seen in the Positive Aging Clinic. One patient was scheduled but did not arrive to their appointment. The mean patient age was 69 years of age (*SD* = 7.7; range 58.9–80.3). Patient-identified gender included male (*n*  *=* 6, 54%) and female (*n*  *=* 5, 45%). Patient self-described race included Black or African American (*n*  *=* 3, 27%), White (*n*  *=* 4, 36%), and unknown/unreported (*n*  *=* 4, 36%). Patient self-described ethnicity included Hispanic or Latino (*n*  *=* 2, 18%), not Hispanic or Latino (*n*  *=* 8, 72%), or unknown/not reported (*n*  *=* 1, 9%). Patient insurance coverage included Medicare (n = 9, 82%), Medicaid (*n* = 4, 36%), or both Medicare and Medicaid (*n*  *=* 3, 27%). Patient employment status included employed (*n*  *=* 1, 9%), retired (*n*  *=* 2, 18%), or unknown (*n*  *=* 8, 72%). Patient-reported use of substances included tobacco (prior cigarette use: *n*  *=* 5, 45%; current cigarette use: *n*  *=* 2, 18%; no tobacco use: *n*  *=* 4, 36%), medical marijuana use (*n*  *=*  *2*, 18%), and current cocaine use (*n*  *=* 1, 9%). No patients reported prior IV (intravenous) drug use, and no patients were uninsured or unhoused. No patients were identified as being members of a vulnerable population. All 11 patients were English-speaking. Additional detailed health data has been omitted to protect patient confidentiality due to the low number of participants. Two faculty geriatricians and one geriatric fellow were involved in the consultative care of these patients. The median number of patient visits with a geriatric provider was 1 (range 1–3).

### Medication Changes

All 11 patients were prescribed ≥10 medications and were also referred for pharmacy consultation. The mean number of prescribed medications was 17.7; *SD* = 7.6; range 10–33. During the pharmacist consultation, two patients had high-risk drug-drug interactions identified. During geriatric consultation, there were 6 episodes of recommending or adding new medications, 6 episodes of recommending alteration to an existing medication, and 18 episodes of recommending discontinuing medications. There were 3 episodes of discontinuation or alteration of medications as recommended by geriatrician (16% of total recommendations) at 5-month follow up. Details of medication changes are described in Table 2.

**Table 2.** Summary of Pharmaceutical Evaluation and Recommendations for Medication changes

Patient	High-Risk Medications By Beers Criteria	Drug-drug Interactions	Medication Added/altered By Geriatrician	Geriatrician Recommended Discontinuing Medication	Geriatrician Recommended Adding Medication	Geriatrician Recommended Altering Medication	Geriatrician Recommended Alternate Agent	5-m Follow Up
1	Diazepam, quetiapine	-	Increased quetiapine, split into twice daily dosing	Provided slow and detailed diazepam taper	Vitamin D, vitamin B12	−	−	Benzodiazepine taper, remains on quetiapine No notes on vitamin supplementation
2	−	-	-	-	-	-	-	-
3	Hydromorphone, cyclobenzaprine	…	…	Hydromorphone, cyclobenzaprine	-	-	-	Patient declined discontinuation of medications
4	Clonazepam, indomethacin, ranitidine, tizanidine	Atazanavir/ritonavir interactions with bupropion, clonazepam, clopidogrel, ranitidine, and vardenafil	-	Controlled clonazepam taper, consider SNRI/SSRI during taper	-	-	-	Patient declined benzodiazepine taper
5	Cyclobenzaprine, gabapentin, hydrocodone-acetaminophen, ibuprofen, pregabalin, tramadol, tizanidine, ziprasidone, zolpidem	-	-	Cyclobenzaprine, gabapentin, hydrocodone-acetaminophen, ibuprofen, pregabalin, tramadol, tizanidine, ziprasidone, zolpidem	-	-	-	Cyclobenzaprine and tizanidine removed from medication list
6	Hydroxyzine	-	-	-	-	Take hydroxyzine at night, switch to less sedating antihistamine if advancing age or falls	-	Changes not noted in chart
7	−	-	-	-	Add SSRI in addition to existing mirtazapine	-	-	Changes not noted in chart
8	−	Etravirine and glipizide, levothyroxine, atorvastatin Darunavir/ritonavir and glipizide, levothyroxine, atorvastatin, loratadine	-	Consider discontinuation of icosapent ethyl	-	-	Combination HIV medication (if pill is not too large) to increase compliance	Changes not noted in chart
9	Ibuprofen, oxybutynin, ranitidine, tramadol, trazodone	-	-	Limit tramadol and trazodone	-	-	Oxybutynin to mirabegron for decreased anticholinergic effects	Changes not noted in chart
10	Eszopiclone, lorazepam, morphine, oxycodone, pregabalin	-	IV iron added	Eszopiclone or lorazepam	SNRI (duloxetine)	Vitamin B12 increased	-	Duloxetine started Patient declined lorazepam discontinuation
11	Ibuprofen	-	-	-	Aspirin	-	Topical lidocaine or diclofenac instead of ibuprofen	Daily aspirin started Patient declined diclofenac gel

Abbreviations: SSRI, selective serotonin reuptake inhibitor; SNRI, serotonin and norepinephrine reuptake inhibitor; IV, intravenous.

### Summary of Topics Addressed During Geriatrics Consultation

All non-pharmacologic interventions performed during consultation visits are detailed in Table 3. Geriatric assessments performed included timed get-up-and-go (*n*  *=* 7, 64%), PHQ-2 (*n*  *=* 5, 45%), Montreal Cognitive Assessment (MOCA) (*n*  *=* 4, 36%), Mini-Cog (*n*  *=* 3, 27%), GAD-7 (*n*  *=* 1, 9%), and grip strength testing (*n*  *=* 1, 9%). Additional interventions included discussion of advanced care planning (*n*  *=* 11, 100%), addition of new diagnoses/problems to patient's problem list (*n*  *=* 5, 45%), referral to other health services (*n*  *=* 4, 36%), and referral to community-based services such as Meals on Wheels (*n*  *=* 3, 27%). Specific recommendations were provided by the geriatrician to the patient or primary HIV provider for osteoporosis screening (*n*  *=* 6, 54%), memory problems (*n*  *=* 5, 45%), mental health (*n*  *=* 5, 45%), hearing loss (*n*  *=* 3, 27%), falls (*n*  *=* 2, 18%), nutrition/weight loss (*n*  *=* 2, 18%), urinary incontinence (*n*  *=* 1, 9%), cancer screening (*n*  *=* 1, 9%), elder abuse (*n*  *=* 1, 9%), and mobility (*n* = 1, 9%). Recommendations included referral for bone mineral density testing or re-initiation of bisphosphonate therapy for osteoporosis, discontinuation of centrally acting medications for memory problems, referral to audiologist or otolaryngology for cerumen disimpaction for hearing loss, referral to vestibular physical therapy or home occupational therapy for falls, referral to social work for weight loss related to food insecurity, and referral to endocrinology for uncontrolled hypothyroidism and diabetes. On chart review at 5 months, 7 geriatrician recommendations related to functional status had been completed (64%); 6 recommendations related to preventative care had been completed (60%); 5 recommendations related to socioenvironmental interventions had been completed (63%); and 6 recommendations related to cognition/mental health had been completed (*n*  *=* 5, 45%) or partially completed (*n*  *=* 1, 9%). Out of 10 provider or community referrals recommended or placed, 7 (70%) had been ordered, scheduled or completed at the time of follow up.

**Table 3.** Summary of Non-pharmaceutical Interventions or Recommendations During Geriatric Consultation and Whether These Were Completed at 5-month Chart Review

Patient	New Diagnosis Added	Referrals Placed	Functional Status Interventions	Preventative Care Interventions	Socioenvironmental Interventions	Cognitive/mental Health Interventions
1	-	-	*Weight loss/nutrition*—optimize mental health regimen, advised against mirtazapine, smoking cessation (partially completed)	*Osteoporosis*—DEXA scan (not completed)	*Advanced care planning*—consider social work consult for state appointed decision maker (not completed)	*Mental health*—increase quetiapine for bipolar disorder, benzodiazepine taper (partially completed)
2	Hearing impairment, mild cognitive impairment	ENT consult (completed)	*Hearing loss*—ENT for cerumen decompaction (completed)	*Osteoporosis*—DEXA scan, check vitamin D (not completed); *Cancer screening*—mammogram (unknown if completed)	-	*Cognition*—check B12, TSH (completed)
3	-	PT vestibular rehabilitation, (completed)	*Mobility*—4-wheel walker (completed) *Falls*—discontinue centrally acting medications, PT (not completed)	-	*Advanced care planning*—recommended Guardianship Alliance (completed)	*Cognition*—consider low dose gabapentin for possible complex seizure activity, discontinue hydromorphone and cyclobenzaprine as could be confounding effects of memantine (not completed)
4	Sleep apnea	Sleep study (completed)	*Hearing loss*—audiologist appointment (completed) *Polypharmacy*—Seniors Clinic or ID pharmacy assistance in tapering meds (unknown if completed)	-	-	-
5	-	Social work consult (completed)	-	-	*Elder abuse*—high risk for financial exploitation, recommended social work consult (completed) *Advanced care planning*—needed prior to surgery, recommended social work consult (completed)	*Mental health*—advised referral to psychiatry for uncontrolled bipolar disorder (completed)
6	-	-	-	*Osteoporosis*—DEXA scan (completed)	*Advanced care planning*—paperwork provided, MOST form completed, online resources provided (paperwork not completed)	-
7	-	Pharmacy to coordinate with home health RN (completed)	-	-	*Advanced care planning*—discussed (paperwork not completed)	*Cognition*—pharmacy to contact home health RN to clarify medication regimen, concern for compliance (completed); formal testing at follow-up visit (not completed)
8	Frailty	Endocrinology consult (unknown if completed), case management (unknown if completed), SW for food insecurity (completed)	*Weight loss/nutrition*—screened for food insecurity (completed)	-	*Food access—*social work consultation (completed)	*Cognition*—repeat testing with cochlear implants (not completed) *Mental health*—recommend addition of SSRI in future if needed for symptom control (unknown if completed)
9	Dizziness, Falls	Home OT safety eval, consider PT for dizziness (completed)	*Hearing loss*—advised hearing test (unknown if completed) *Incontinence—*continue following with urology, consider switch from oxybutynin to mirabegron (not completed) *Falls*—PT/OT evaluation (not completed)	*Osteoporosis—*DEXA scan (not completed)	-	-
10	-	-	-	*Osteoporosis*—DEXA scan (completed)	-	*Mental health*—recommended SNRI (completed)
11	Prostate cancer	…	*Hearing loss*—advised hearing test	*Osteoporosis*—DEXA scan (completed) *Cancer screening*—colonoscopy (completed), low dose CT chest (completed), *Other screening*—AAA screening ultrasound (completed)	*Social isolation*—discussed involvement in community groups (completed)	*Mental health*—therapy and substance abuse counselor (completed) *Cognition*—formal memory test at follow-up visit (not completed)

Blank spaces (denoted “-“) indicate that the relevant intervention or recommendation was not applicable or was deferred by geriatric provider according to clinical judgement.

Abbreviations: DEXA, dual energy X-ray absorptiometry; ENT, ear, nose, and throat; TSH, thyroid-stimulating hormone; PT, physical therapy; OT, occupational therapy; ID, infectious disease; MOST, Medical Orders for Scope of Treatment; SSRI, selective serotonin reuptake inhibitor; SNRI, serotonin and norepinephrine reuptake inhibitor; CT, computed tomography; MoCA, Montreal Cognitive Assessment; AAA, abdominal aortic aneurysm.

### Primary Provider Satisfaction

**Figure 1.**
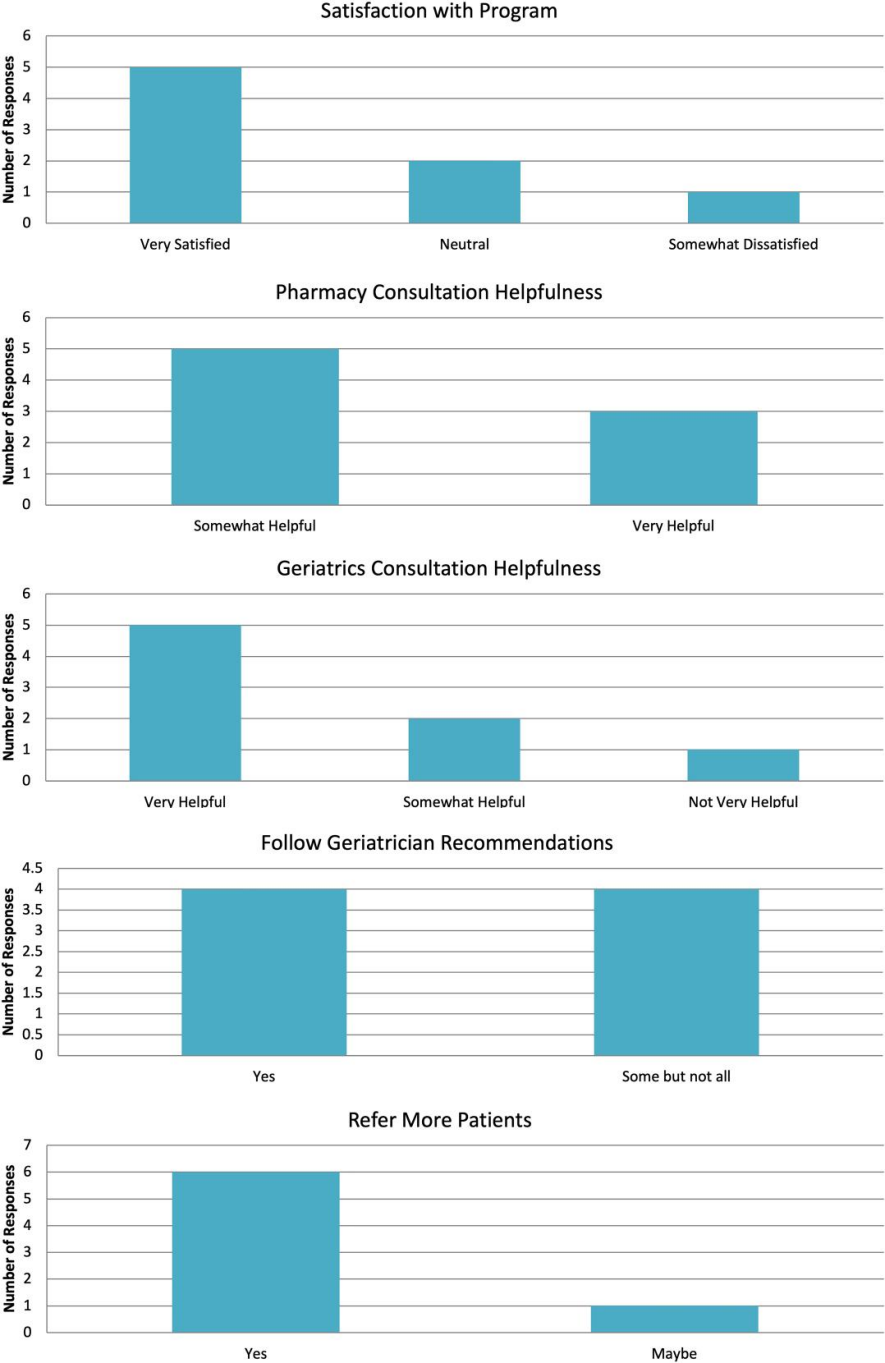
Summary of HIV providers' survey responses. caption: Summary of HIV providers' responses (*n* = 9) regarding their experiences with the geriatric consultation program. Bars represent the number of providers selecting each response across five domains: overall satisfaction with the program, perceived helpfulness of pharmacy and geriatrics consultations, likelihood of following geriatrician recommendations, and willingness to refer additional patients. Providers generally reported high satisfaction, found consultations helpful, and indicated strong intent to implement recommendations and refer future patients.

Responses were recorded from HIV providers regarding the experience of each patient they referred for geriatric consultation. A total of 8 surveys were completed from 6 unique providers (1 infectious disease nurse practitioner, 5 infectious disease physicians). The responses are summarized in Figure 1. Overall, providers reported being very satisfied (*n*  *=* 5, 56%), neutral (*n*  *=* 2, 22%), or somewhat dissatisfied (*n*  *=* 1, 11%) with the program. Providers found the pharmacy consultation to be very helpful (*n*  *=* 2, 22%) or somewhat helpful (*n*  *=* 4, 44%), and the geriatrics consultation to be very helpful (*n*  *=* 5, 56%), somewhat helpful (*n*  *=* 2, 22%), or not very helpful (*n*  *=* 1, 11%). Almost all providers reported that they would follow-through with geriatrician recommendations (“yes” [*n* = 4, 44%]; “some, but not all” [*n*  *=* 4, 44%]), and almost all providers reported that they would refer more patients for Positive Aging Consultation (“yes” [*n*  *=* 7, 78%]; “maybe” [*n*  *=* 1, 11%]). Narrative comments noted satisfaction with quick appointment times and the identification of several new screenings for a patient. One provider noted that their patient enjoyed the experience but is generally resistant to changes to their medications. One provider wished they had been provided more specific recommendations for medication tapering.

## DISCUSSION

The Positive Aging Consultation program resulted in 11 people with HIV from the UCHealth Infectious Disease Clinic receiving outpatient consultation from a clinical pharmacist and a geriatric specialist. The program supported a dedicated medication review and access to specialist care for patients who would typically have limited access to geriatric evaluation, based on the age cutoff of 75 years at the Seniors Clinic (80% < 75 years) [9]. Patients benefitted from longer appointment times than a typical HIV clinic visit, which allowed for identification of high-risk medications, referrals to other specialists or services, and recommendations for issues related to functional status, preventative care, socioenvironmental factors, and cognitive concerns/mental health. Providers were satisfied overall with the program and likely to refer patients in the future. Despite these positive outcomes, the most notable outcome of this study was the overall limited utilization of the consultation service, which prompted us to consider barriers to geriatric consultation in people with HIV.

One barrier to implementation of the geriatric consultation model may be the lack of patient and provider awareness of or consensus on the utility of geriatric consultation, particularly in patients who may not self-identify as “geriatric”. The geriatric consultation program represented a comprehensive geriatric assessment (CGA), which can be defined as a multidimensional, multidisciplinary assessment of an older person's functional ability, physical health, cognition, and socioenvironmental circumstances [21]. Studies have shown that CGAs have been linked to improved survival, connection to care, functional status, and quality of life, as well as decreased caregiver burden and hospital readmission rates in older adults [22–25]. Various specialties and subspecialties, including surgery, cardiology, nephrology, and oncology, have shown improvement in outcomes and quality of life with the implementation of CGAs [26–34]. In fact, CGA was shown to be so beneficial in decreasing chemotherapy-related toxicity, surgical complications, and poor functional status in oncology patients that the American Society of Clinical Oncology guidelines now recommend CGA for all older patients undergoing cancer treatment [35].

Regarding CGA for HIV, existing guidelines address components of management of the aging HIV population. In 2011, the American Geriatrics Society, the American Academy of HIV Medicine, AIDS Community Research Initiative of America jointly released the first guidelines for treatment of older HIV patients [36]. Since then, the Department of Health and Human Services, the International Antiviral Society-USA, and the Infectious Diseases Society of America have added sections on caring for aging people with HIV to their guidelines [37–39]. Implementation of geriatric care for HIV patients, however, remains limited, and a gold-standard model of care for this population has not yet been defined [7, 8].

Because we only conducted surveys of referring providers, we do not know exact reasons why some HIV providers did not participate in the program. We suspect that the lack of an integrated, co-located clinic for geriatric care was another barrier. Despite the Seniors Clinic being in the same building as the Infectious Disease clinic, the need for a separate referral and establishing care in a new clinic likely served as a deterrent to participation in the program. Furthermore, providers often forgot that the consultation service existed without the regular presence of a geriatrician in the clinic. Indeed, a systematic review found that co-located models of HIV and non-HIV medical care had improved linkage to care and antiretroviral therapy outcomes compared to other models [40]. Integrated, multidisciplinary care has been associated with decreased hospitalizations, reduced duplicate testing, and lower healthcare costs for people with HIV [41]. Furthermore, a 2023 survey of people with HIV in the University of Colorado Infectious Disease clinic found that less than half (46%) of responders would be very likely to see a provider that specializes in aging, while 61% would be very likely, if the provider was located within the HIV clinic itself [42]. As such, future models may benefit from a co-located approach to geriatric care to improve patient and provider participation. Notably, with the increased prevalence of telehealth spurred by the COVID-19 pandemic, internet-based models of care may potentially be utilized to optimize delivery of integrated care [43].

We suspect that success in de-prescribing would have driven more referrals. Polypharmacy is commonly encountered among older adults with HIV and contributes to increased risks of falls and frailty [38]. Hyper-polypharmacy was present in all people referred for geriatric consultation in our clinic setting and many medication discontinuation or alteration recommendations were made. However, patients frequently declined these changes: only two medications were removed from a medication list after 5 months, and one benzodiazepine taper was ongoing. This lack of follow-through with geriatric recommendations has been echoed in other studies of HIV patients. For example, a retrospective cohort study of a geriatric consultation model at a New York HIV clinic cited only 32.4% adherence to geriatrician recommendations regarding medications [44]. Multiple systematic reviews have described a variety of barriers to successful medication de-prescribing including fragmented care among multiple providers, high clinical complexity, time restraints, and incomplete information regarding rationales for prescription of certain drugs [45–47]. Many providers lack training or familiarity with tapering and monitoring medications, or may fear potential consequences of de-prescribing, such as withdrawal or undertreatment [48]. Developing clear guidance and demonstrating effective de-prescribing may improve perceived benefits of geriatric consultation.

A major barrier to implementing geriatric care for people with HIV within any healthcare system is the national shortage of geriatricians. The Health Resources and Services Administration predicts that there will be a 50% increase in demand for geriatricians between 2018–2030 in the U.S., and the supply of geriatricians will decrease slightly in that time [49]. As the population ages, access to specialized geriatric care will be difficult even for people ≥75 years of age [49–51]. A thorough geriatric assessment is time-consuming for consulting providers and pharmacists, and many geriatric clinics may lack the ability to expand their patient population to include regular subspecialty consultations [50]. Because of the limited access to specialty geriatric care, all clinicians will need to develop basic skills in how to care for older adults [51, 52]. Dedicated training in geriatric care for HIV providers, or HIV certification for geriatric providers, may be an effective, cost-effective, and efficient way to expand geriatric care to people with HIV [7].

Lastly, financial concerns are often considered a major barrier in implementing geriatric consultation in many settings. Indeed, a barrier for co-location in the present study was the inability for the geriatrician to bill if physically seeing a patient within the HIV clinic. Any visits outside of the geriatric clinic were essentially volunteered [7]. Geriatricians are often viewed by insurers as primary care providers rather than consulting specialists, which may cause confusion around reimbursement and coverage [7, 53, 54]. For instance, the Centers for Medicare & Medicaid Services may not provide reimbursement if a patient has multiple visits for the same services in a single day [7]. Despite multiple benefits of integrated care as discussed above, billing regulations may prevent patients from seeing multiple specialists (ie, infectious disease and geriatrics) in one single integrated clinic [54]. Potential solutions to these financial constraints include the ability to reimburse specialty care for patients who lack adequate insurance coverage, training members of the HIV clinic team to perform some or all of the CGA as part of usual HIV care, or establishing practice agreements that allow geriatricians to bill as an extension of the HIV care team.

Limitations of our study include a small sample size and a single clinical site, which limits generalizability. The follow-up time was 5 months, which is likely insufficient to draw significant conclusions regarding patient outcomes. The study also lacks robust data regarding patients' perspectives on their experience with geriatric consultation, which will be a valuable component to consider in future studies and has been collected in a separate study [42]. However, we provide a detailed account of clinical interventions resulting from geriatric evaluation and reveal limitations to the clinical model in real-world practice. Barriers to geriatric care in our setting highlight opportunities for improvement in future care models.

## CONCLUSION

The Positive Aging Consultation program served as an initial step towards addressing the needs of the aging population of people with HIV in the University of Colorado Infectious Disease Clinic. Despite limited participation of patients and providers, we identified key barriers that face implementation of geriatric care for people with HIV. Recognition of these barriers is essential to designing more scalable models of care moving forward. Future efforts should focus on strategies to mitigate these barriers, which may include educational efforts surrounding the benefits of comprehensive geriatric assessments, more integrated and inclusive clinical models, and working with institutions and insurance to ensure available funding for these endeavors.
